# Long non-coding RNA *TUSC7* acts a molecular sponge for miR-10a and suppresses EMT in hepatocellular carcinoma

**DOI:** 10.1007/s13277-016-4892-6

**Published:** 2016-03-22

**Authors:** Yufeng Wang, Zhikui Liu, Bowen Yao, Changwei Dou, Meng Xu, Yumo Xue, Linglong Ding, Yuli Jia, Hongyong Zhang, Qing Li, Kangsheng Tu, Yang Jiao, Qingguang Liu, Cheng Guo

**Affiliations:** 1 Department of Hepatobiliary Surgery, First Affiliated Hospital of Medical College of Xi’an Jiaotong University, Xi’an, 710061 Shaanxi China; 2 Department of Prosthodontics, State Key Laboratory of Military Stomatology, School of Stomatology, The Fourth Military Medical University, Xi’an, China; 3 Shaanxi Key Laboratory of Military Stomatology, Xi’an, Shaanxi China

**Keywords:** *TUSC7*, miR-10a, *EphA4*, EMT, HCC

## Abstract

Despite advances in the roles of long non-coding RNA (lncRNA) tumor suppressor candidate 7 (*TUSC7*) in cancer biology, which has been identified as a tumor suppressor by regulating cell proliferation, apoptosis, migration, invasion, cell cycle, and tumor growth, the function of *TUSC7* in hepatocellular carcinoma (HCC) remains unknown. In this study, we observed that the expression of *TUSC7* was immensely decreased in HCC. Clinically, the lower expression of *TUSC7* predicted poorer survival and may be an independent risk factor for HCC patients. Moreover, *TUSC7* inhibited cell metastasis, invasion, and epithelial-to-mesenchymal transformation (EMT) through competitively binding miR-10a. Furthermore, we found that *TUSC7* could decrease the expression of Eph tyrosine kinase receptor A4 (*EphA4*), a downstream target of miR-10a as well as an EMT suppressor, through *TUSC7*-miR-10a-*EphA4* axis. Taken together, we demonstrate that *TUSC7* suppresses EMT through the *TUSC7*-miR-10a-*EphA4* axis, which may be a potential target for therapeutic intervention in HCC.

## Introduction

Hepatocellular carcinoma (HCC) is the most common type of primary liver cancer and the second leading cause of death from cancer worldwide [1]. Currently, the prognosis for HCC patients remains poor, with a 5-year survival rate of approximately 30 % after liver resection, which is considered to be the best therapeutic strategy to treat HCC [2]. Local and systemic metastases are the main reasons for the unsatisfactory prognosis of HCC patients [3]. Elucidating the underlying molecular mechanisms for HCC metastasis is critical for identifying novel therapeutic targets of HCC.

Epithelial-to-mesenchymal transformation (EMT) has been widely accepted as a key mechanism underlying the metastatic process of HCC [4]. During the development of EMT, the expressions of epithelial markers such as E-cadherin, zonula occludens-1, and claudins decrease while the expressions of mesenchymal markers such as vimentin, N-cadherin, and fibronectin increase [5]. The EMT process in HCC cells can be regulated by various factors, including hypoxia [6], cytokines [7], long non-coding RNAs (lncRNAs) [8], microRNAs [9], and so on, and targeting the EMT process has been found to be an attractive and promising strategy to prevent the metastasis of HCC [7].

lncRNAs are RNA molecules over 200 nucleotides in length with little protein-coding potential [10]. Previous studies have shown that aberrant lncRNA expression is observed in human cancers, including those in the liver [11], breast [12], colon [13], ovary [14], pancreas [15], and bladder [16]. lncRNAs have been identified with oncogenic properties (*KRASP*, *HULC*, *HOTAIR*, *MALAT1*, *HOTTIP*, *ANRIL*, and *RICTOR*) or oncosuppressive properties (*MEG3*, *GAS5*, *LincRNA-p21*, *PTENP1*, *TERRA*, *CCND1*/*CyclinD1*, and *TUG1*) or both (*CCAT1* and *XIST*) [17, 18]. Tumor suppressor candidate 7 (*TUSC7*), also called *LOC285194* or *LSAMP* antisense RNA3, is an lncRNA consisting of four exons of more than 2 kb in length and is located at 3q13.31 [19]. Recent studies indicated that lncRNA *TUSC7* is downregulated in cancers including gastric cancer [20], osteosarcoma [21], colorectal cancer (CRC) [22], esophageal squamous cell carcinoma (ESCC) [23], and so on. In gastric cancer, *TUSC7* is a p53-regulated tumor suppressor that acts in part by repressing miR-23b to suppress tumor cell growth in vitro and in vivo [20]. In osteosarcoma, depleting *TUSC7* promoted proliferation of normal osteoblasts by regulating apoptotic and cell cycle transcripts as well as the vascular endothelial growth factor (VEGF) receptor 1 [21]. In human pancreatic ductal adenocarcinoma (PDAC) and CRC, by analyzing the association of *TUSC7* expression with clinicopathologic features, it was found that low *TUSC7* expression was closely correlated with lymph node metastasis, liver metastasis, and more distant metastases [19, 22]. These data validated that *TUSC7* is a tumor suppressor by regulating cell proliferation, apoptosis, migration, invasion, cell cycle, and tumor growth. However, the exact role of *TUSC7* in HCC progression and the underlying mechanisms remain unknown.

MicroRNAs (miRNAs) are an abundant group of endogenous non-coding single-strand RNAs, and it is known that aberrant miRNA expression profiles are causally connected to tumor progression [24]. Recently, the competing endogenous RNA (ceRNA) hypothesis proposed that a large number of non-coding RNAs might function as molecular sponges for miRNAs and, hence, functionally liberate other RNA transcripts targeted by the aforementioned active miRNAs [25]. For example, lncRNA-*UCA1* has been reported to play an oncogenic role in breast cancer through directly interacting with miR-143 to lower its expression and affect its downstream regulation [26]. miR-222 could be downregulated by lncRNA-Gas5 in glioma, thereby suppressing the tumor malignancy [27, 28], and has been reported to play critical roles in the development of a variety of human cancers [29–31], including HCC [28, 32]. In HCC, acting as a tumor promoter, the expression of miR-10a has been shown to be upregulated, which accelerates the cell migration, invasion, and EMT [32, 33]. Additionally, Eph tyrosine kinase receptor A4 (*EphA4*), a member of the Eph receptor tyrosine kinase family, has been identified as an EMT suppressor in cancers [34–36]. It is reported that miR-10a could regulate the EMT process in HCC through directly binding the 3′-untranslated region (UTR) of the *EphA4* transcript [32]. However, limited knowledge is available concerning whether *TUSC7* could act as a sponge for miR-10a to affect the biological processes of HCC and the potential primary mechanism among *TUSC7*, miR-10a, and *EphA4* in HCC progression remains unknown.

In this study, we found that the expression of *TUSC7* was decreased in HCC and that *TUSC7* may be a promising prognostic or progression marker for HCC. Additionally, *TUSC7* suppressed cell migration, invasion, and EMT of HCC cells. Moreover, mechanistic analysis revealed that *TUSC7* may function as a ceRNA for miR-10a to regulate the expression of *EphA4* to suppress EMT in HCC, thus playing an oncosuppressive role in HCC pathogenesis. Here, we provide the first evidence for the *TUSC7*-miR-10a-*EphA4* axis, shedding new light on the mechanism of HCC.

## Materials and methods

### Clinical samples

HCC samples were collected from 75 patients including 51 males and 24 females, who underwent resection of their primary HCC in the Department of Hepatobiliary Surgery at the First Affiliated Hospital of Xi’an Jiaotong University during January 2009 to December 2011. Patients did not receive any preoperative chemotherapy or embolization.

Patients’ demographic and clinicopathologic data were obtained through a review of hospital records. And disease recurrence and survival information was updated at each follow-up visit. The time between the surgery date and first disease recurrence date was calculated as disease-free survival (DFS). The time between the diagnostic biopsy and surgery date to death or last follow-up was determined as overall survival (OS) duration.

### Cell culture

The human immortalized normal hepatocyte cell line (LO2) and six HCC cell lines (HepG2, MHCC97L, Hep3B, SMMC-7721, MHCC97H, and Huh7) were obtained from the Institute of Biochemistry and Cell Biology, Chinese Academy of Sciences, Shanghai, China. All cells were cultured in complete Dulbecco’s modified Eagle’s medium (DMEM; Gibco, Grand Island, NY, USA) containing 10 % fetal bovine serum (FBS; Gibco) with 100 units/mL penicillin and 100 μg/mL streptomycin (Sigma, St. Louis, MO, USA) in a humidified incubator containing 5 % CO_2_ at 37 °C.

### Cell transfection

Three *TUSC7*-specific small interfering RNAs (siRNAs), the *TUSC7*-siControl (Table 1), pcDNA3.1-*TUSC7* (pcDNA/*TUSC7*), and pcDNA3.1-Control (pcDNA/Control), were purchased from Invitrogen (Carlsbad, CA, USA). Four miRNA vectors, including anti-miR-10a, anti-Control, miR-10a, and miR-10a-Control, were purchased from GeneCopoeia (Guangzhou, China). All cell transfections were performed according to the manufacturer’s protocol.

**Table 1:** *TUSC7*-siRNAs and *TUSC7*-siControl sequences

siRNA	Sequence
*TUSC7*-siRNA1	Sense: 5′-GGCCAAACCCUCAAUGAAUtt-3′ Antisense: 5′-AUUCAUUGAGGGUUUGGCCtg-3′
*TUSC7*-siRNA2	Sense: 5′-GCGCAUUUCUCUUAAACAATT-3′ Antisense: 5′-UUGUUUAAGAGAAAUGCGCTT-3′
*TUSC7*-siRNA3	Sense: 5′-CUGCCCUCCAUUCUAUCUATT-3′ Antisense: 5′-UAGAUAGAAUGGAGGGCAGTT-3′
*TUSC7*-siRNA4	Sense: 5′-GGAGAGAGAUAUGCUAAGUTT-3′ Antisense: 5′-ACUUAGCAUAUCUCUCUCCTT-3′
*TUSC7*-siControl	Sense: 5′- UUCUCCGAACGUGUCACGUTT-3′ Antisense: 5′-ACGUGACACGUUCGGAGAATT-3′

### Luciferase reporter assay

To search for the miR-10a binding site of *TUSC7*, we used a number of bioinformatics tools (MicroRNA, Mircode, Starbase v2.0, and RNAhybrid). The putative miR-10a target binding sequence in *TUSC7* and its binding site mutant were synthesized and cloned downstream of the luciferase gene in the pmirGLO luciferase vector (Promega, Madison, WI, USA). Hep3B cells were co-transfected with wild-type or mutated pmirGLO-miR-10a reporter plasmid and pcDNA/Control or pcDNA/*TUSC7* using Lipofectamine 2000 (Invitrogen). After 48 h, the cells were harvested and luciferase activity was measured using the dual-luciferase reporter assay system (Promega, Madison, WI, USA). Firefly luciferase activity was normalized to the Renilla luciferase activity. Results were obtained from three independent experiments performed in triplicate.

### RNA extraction and quantitative real-time PCR

Total RNA was extracted from HCC tissues and cell lines using TRIzol (Invitrogen) following the manufacturer’s instructions. The RNA levels of *TUSC7* and *EphA4* were determined by quantitative real-time PCR (qRT-PCR) and calculated using the 2^−ΔΔCt^ method, with the Ct values normalized using GAPDH as an internal control. The primers are listed in Table 2. miRNAs were obtained using the mirVana MiRNA Isolation Kit (Ambion, Austin, TX, USA). Mature miR-10a and *U6* snRNA were reversely transcribed using Stem-loop RT Primer with miScript II RT Kit (Qiagen, Valencia, CA, USA). qRT-PCR was performed using SYBR Green PCR Master Mix (Qiagen) in an ABI 7500 system (Applied Biosystems, USA).

**Table 2:** Primers used in qRT-PCR

Primer name	Sequence (5′–3′)
GAPDH	Forward: 5′-CCGGGAAACTGTGGCGTGATGG-3′ Reverse: 5′-AGGTGGAGGAGTGGGTGTCGCTGTT-3′
*TUSC7*	Forward: 5′- CACTGCCTATGTGCACGACT-3′ Reverse: 5′- AGAGTCCGGCAAGAAGAACA-3′
E-cadherin	Forward: 5′- GCCGCTGGCGTCTGTAGGAA -3′ Reverse: 5′- TGACCACCGCTCTCCTCCGA -3′
Vimentin	Forward: 5′-GAGAACTTTGCCGTTGAAGC-3′ Reverse: 5′-GCTTCCTGTAGGTGGCAATC-3′
*EphA4*	Forward: 5′ - ATGGATCCTGTTGCCCTCAC -3′ Reverse: 5′- CAGAATTCCTCCTACCCTTACC -3′

### Western blot

Western blot analysis was performed using standard techniques. The following antibodies were used: E-cadherin (3195S, Cell Signaling, Beverly, MA, USA), vimentin (sc-6260, Santa Cruz Biotechnology, Santa Cruz, CA, USA), *EphA4* (SRP00347b, Saierbio, Tianjin, China), and β-actin (sc-47778, Santa Cruz Biotechnology, Santa Cruz, CA, USA).

### Wound healing assays

To determine cell motility, HCC cells were seeded into six-well plates and grown to 80–90 % confluence. A 200-μL sterile plastic tip was used to create a wound line across the surface of plates, and cellular debris was removed by washing with phosphate-buffered saline (PBS). Cells were cultured in DMEM in a humidified incubator with 5 % CO_2_ at 37 °C for 48 h, and then images were taken with a phase-contrast microscope.

### Transwell assays

The 8 μM pore-size transwell inserts (Nalge Nunc, Penfield, New York, NY, USA) were coated with Matrigel (BD Biosciences, Franklin Lakes, NJ, USA) at 1:8 dilution on the inner layer. Hep3B and MHCC97H cells were resuspended with reduced serum DMEM, and the density was adjusted to 2.5 × 10^5^/mL 48 h after transfection. A 200-μL cell suspension was added into the upper chamber, and 750 μL DMEM containing 10 % FBS was added into the lower chamber and then incubated for 24 h.

Cells were fixed in 4 % paraformaldehyde for 2 min and then permeabilized in 100 % methanol for 20 min. The cells on the inner layer were softly removed with a cotton swab, and the adherent cells on the undersurface of the insert were stained with 0.3 % crystal violet dye for 15 min. The filters were washed with PBS, and images were taken. Cells on undersurface were counted under a light microscope.

### Immunohistochemistry

Immunohistochemistry staining was performed on paraformaldehyde-fixed paraffin sections. The sections were dewaxed and dehydrated. Following rehydration and antigen retrieval in citrate buffer, endogenous peroxidase activity was blocked for 10 min using 3.0 % hydrogen peroxide. The sections were blocked for 30 min using 10 % goat plasma and then separately incubated with the primary antibodies directed against E-cadherin (1:400) and vimentin (1:200) at 4 °C overnight. The primary antibody was detected using biotinylated secondary antibodies (Golden Bridge Biotechnology, Zhongshan, China) according to the manufacturer’s recommendations. The sections were visualized with diaminobenzidine and counterstained with hematoxylin and then dehydrated in alcohol and xylene and mounted onto glass slides.

### Statistical analysis

Results are presented as mean ± SD. The SPSS statistical package for Windows version 13 (SPSS, Chicago, IL, USA) and GraphPad Prism 5 software (GraphPad Software, Inc., San Diego, CA, USA) were used for the Pearson chi-square test, a two-tailed Student’s *t* test, a Kaplan-Meier plot, a log-rank test, or an ANOVA where appropriate. Differences were considered to be significant when *p* < 0.05.

## Results

### The expression of *TUSC7* was decreased in HCC

**Fig. 1:**
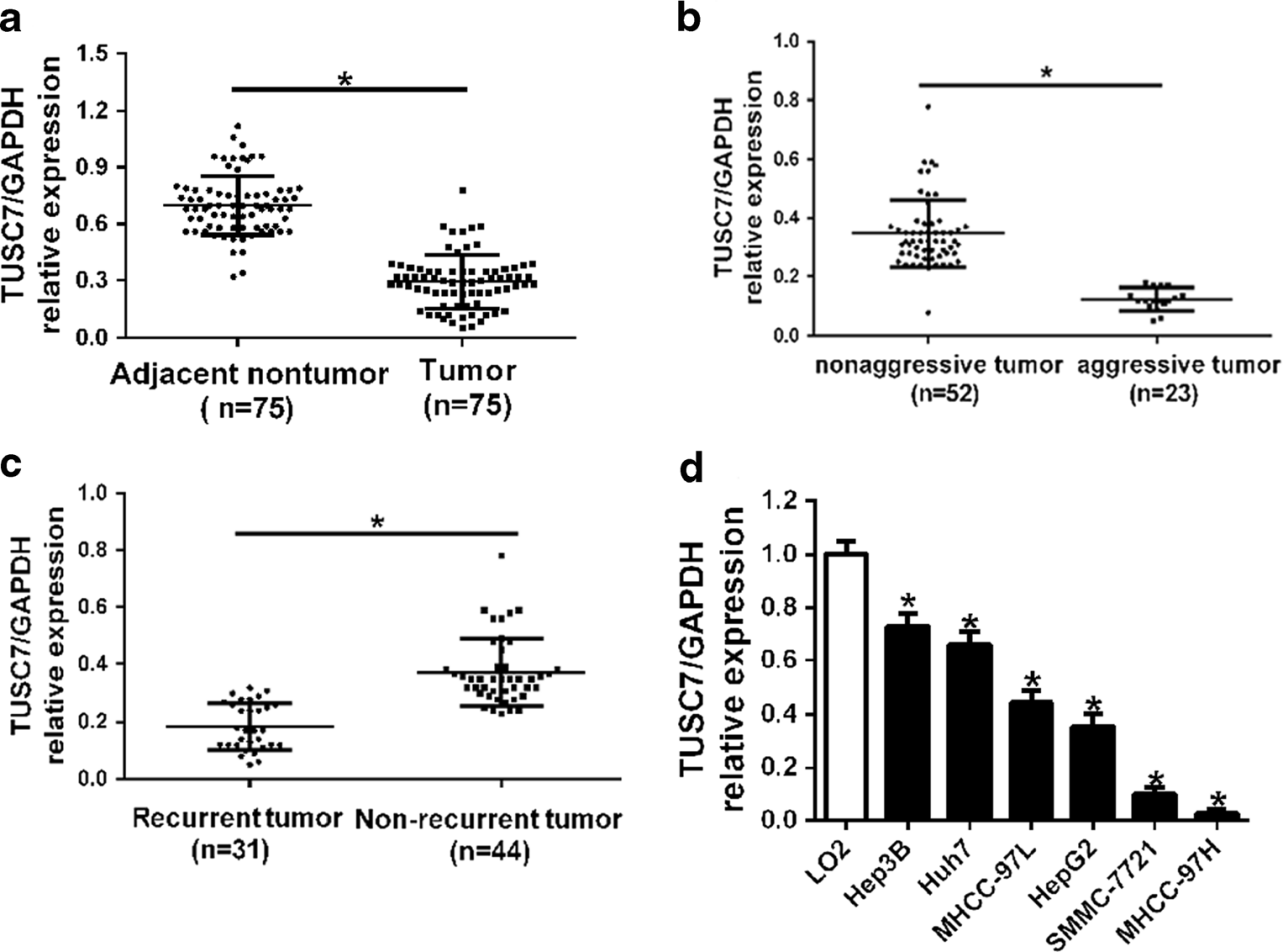
The expression levels of *TUSC7* in HCC. Comparing differences in the expression levels of *TUSC7* between **a** HCC and matched non-tumor tissues, **b** aggressive and non-aggressive tumor tissues, **c** HCC tissues arising from recurrent and non-recurrent groups, and **d** HCC cell lines and the immortalized hepatic cell line LO2. Values are depicted as mean ± SD; *p < 0.05, by *t* test

First, we examined the lncRNA *TUSC7* expression level in 75 paired HCC tissues and adjacent non-tumor tissues by qRT-PCR and normalized them to GAPDH. Our results showed that *TUSC7* levels were significantly decreased in HCC tissues compared with adjacent non-tumor tissues (*p* < 0.05, Fig. 1a). HCC cases with at least one of the clinicopathological features, including intrahepatic spreading, venous infiltration, or tumor invasion, tend to be considered as aggressive HCC tissues. When compared with non-aggressive HCC tissues, *TUSC7* levels were markedly downregulated in aggressive HCC tissues (*p* < 0.001, Fig. 1b). Furthermore, *TUSC7* levels were notably lower in tumor tissues arising from patients with tumor recurrence than that without tumor recurrence (*p* < 0.001, Fig. 1c). Then, expression levels of *TUSC7* in HCC cells were determined by qRT-PCR. Our experiments showed that *TUSC7* expression was significantly downregulated in all HCC cell lines when compared with that in LO2 cells (*p* < 0.05, Fig. 1d). These data suggest that *TUSC7* was frequently downregulated in HCC, especially in those patients with metastases and recurrence, suggesting that *TUSC7* might be associated with migration and metastasis of HCC cells.

### Clinical significance of *TUSC7* expression in HCC

To determine whether *TUSC7* expression is associated with clinicopathological features in HCC patients, HCC patients were divided into two different groups according to the median level of *TUSC7* expression. Further analysis showed that the expression level of *TUSC7* was significantly correlated with tumor nodes (*p* < 0.001), venous infiltration (*p* = 0.017), Edmondson-Steiner grading (*p* = 0.003), and tumor-node-metastasis (TNM) tumor stage (*p* = 0.004) (Table 3). Thus, our results demonstrated that the reduced expression of *TUSC7* was correlated with poor prognostic features of HCC.

**Table 3:** Correlation between the clinicopathologic characteristics and expression of *TUSC7* in HCC

Characteristics	Total no. of patients (*n* = 75)	No. of patients		*p*
		*TUSC7* ^high^	*TUSC7* ^low^	
Age (year)				
<50	19	8	11	0.362
≥50	56	29	27	
Gender				
Male	51	27	24	0.466
Female	24	10	14	
HBV				
Absent	55	26	29	0.346
Present	20	11	19	
Serum AFP level (ng/mL)				
<400	18	7	11	0.309
≥400	57	30	27	
Tumor size (cm)				
<5	43	22	21	0.366
≥5	32	13	19	
No. of tumor nodes				
1	40	28	12	<0.001***
≥2	35	9	26	
Cirrhosis				
Absent	55	28	27	0.651
Present	20	9	11	
Venous infiltration				
Absent	54	22	32	0.017*
Present	21	15	6	
Edmondson-Steiner grading				
I + II	49	18	31	0.003**
III + IV	26	19	7	
TNM tumor stage				
I + II	43	15	28	0.004**
III + IV	32	22	10	

*HBV* hepatitis B virus, *AFP* alpha-fetoprotein, *TNM* tumor-node-metastasis

*p < 0.05; **p < 0.01; ***p < 0.001

**Fig. 2:**
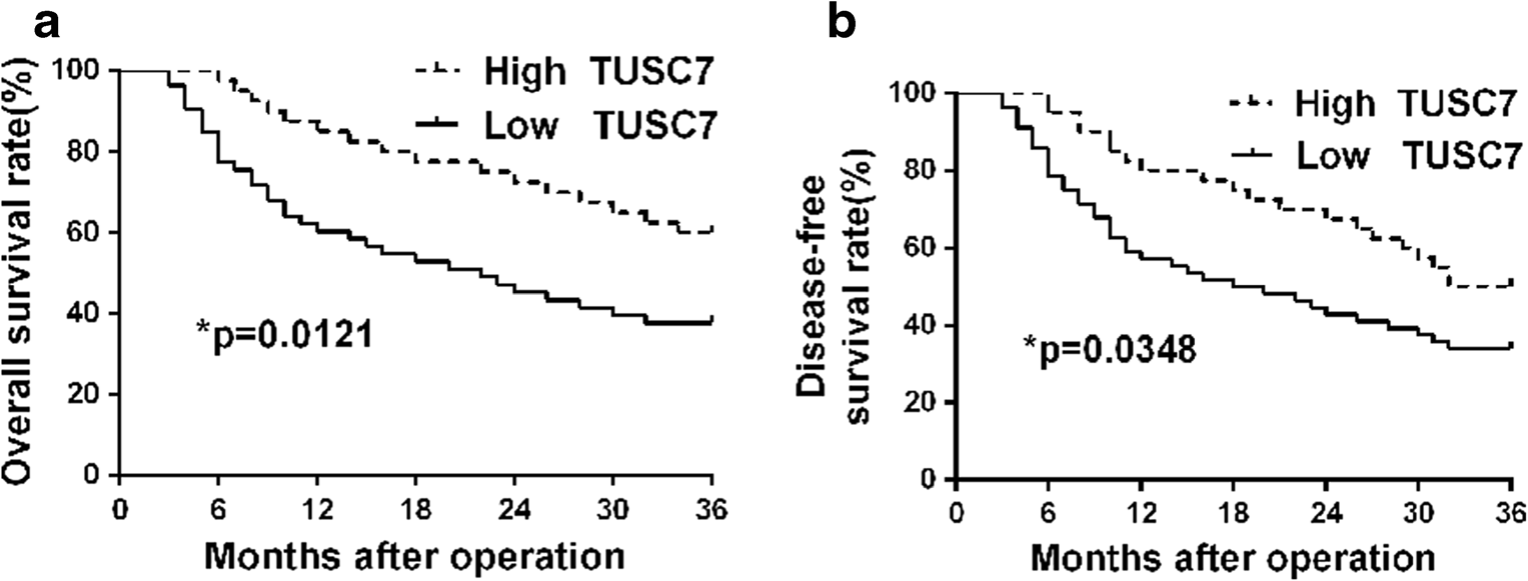
Prognostic significance of *TUSC7* in HCC cases. Kaplan-Meier 3-year **a** overall and **b** disease-free survival curves of HCC patients according to the level of *TUSC7* expression. The low *TUSC7* group (≤0.33, *n* = 53); the high *TUSC7* group (>0.33, *n* = 22). The mean expression value (0.33) obtained for *TUSC7* of the 75 HCC samples detected by qRT-PCR was chosen as the cutoff value. *p < 0.05, by log-rank test

Kaplan-Meier survival curves further revealed that patients with lower *TUSC7* expression had a significantly reduced OS and DFS than those with high *TUSC7* expression (*p* < 0.05, respectively, Fig. 2a, b). Moreover, multivariate Cox proportional hazard regression analysis indicated that venous infiltration and *TUSC7* expression were independent prognostic factors for predicting both 3-year OS and DFS in HCC patients (*p* = 0.007 and 0.015, respectively, Table 4). The data implied that *TUSC7* may be a promising prognostic or progression marker for HCC.

**Table 4:** Multivariate Cox regression analysis of 3-year overall and disease-free survival of 75 HCC patients

Variables	Overall survival			Disease-free survival		
	HR	95 % CI	*p*	HR	95 % CI	*p*
No. of tumor nodules	0.801	0.347, 1.853	0.605	0.598	0.256, 1.400	0.236
Venous infiltration	0.259	0.101, 0.665	0.005**	0.303	0.121, 0.759	0.011*
TNM tumor stage	1.105	0.278, 4.399	0.887	1.089	0.280, 4.230	0.902
*TUSC7* expression	3.411	1.392, 8.357	0.007**	2.928	1.227, 6.985	0.015*
Edmondson-Steiner grading	1.905	0.837, 4.338	0.125	1.660	0.727, 3.792	0.229

*HR* hazard ratio, *CI* confidence interval

*p < 0.05; **p < 0.01

### *TUSC7* inhibits the migration and invasion of HCC cells

**Fig. 3:**
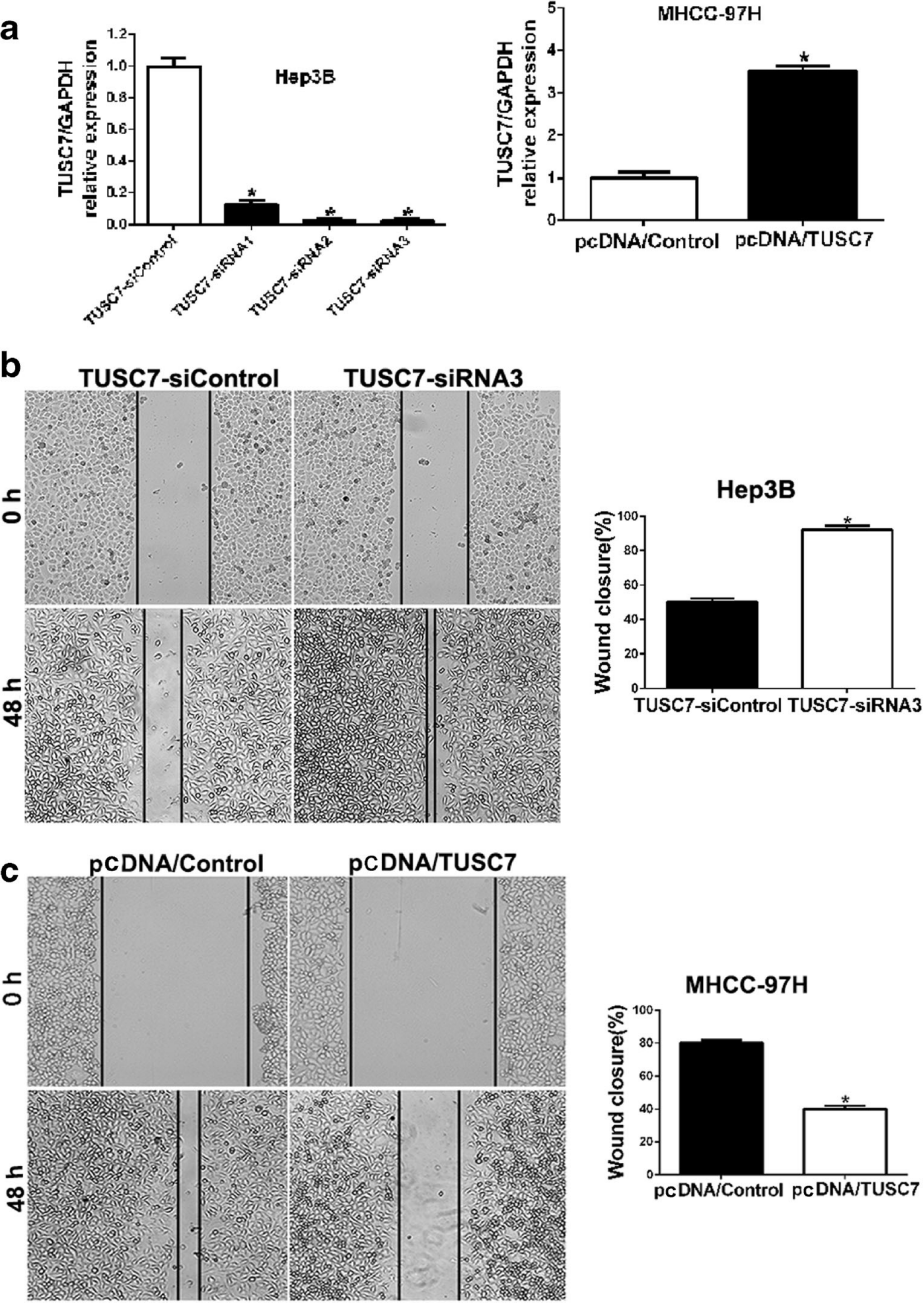
Wound healing assays to assess the effect of *TUSC7* on cell mobility. **a** qRT-PCR analysis revealed that *TUSC7* expression in Hep3B cells was reduced most obviously by *TUSC7*-siRNA3 and pcDNA/*TUSC7* largely increased the *TUSC7* expression in MHCC97H cells. **b** Wound healing assays to assess the effect of *TUSC7* on cell mobility in Hep3B cells. **c** Wound healing assays to assess the effect of *TUSC7* on cell mobility in MHCC97H cells.*P < 0.05, by *t* test

**Fig. 4:**
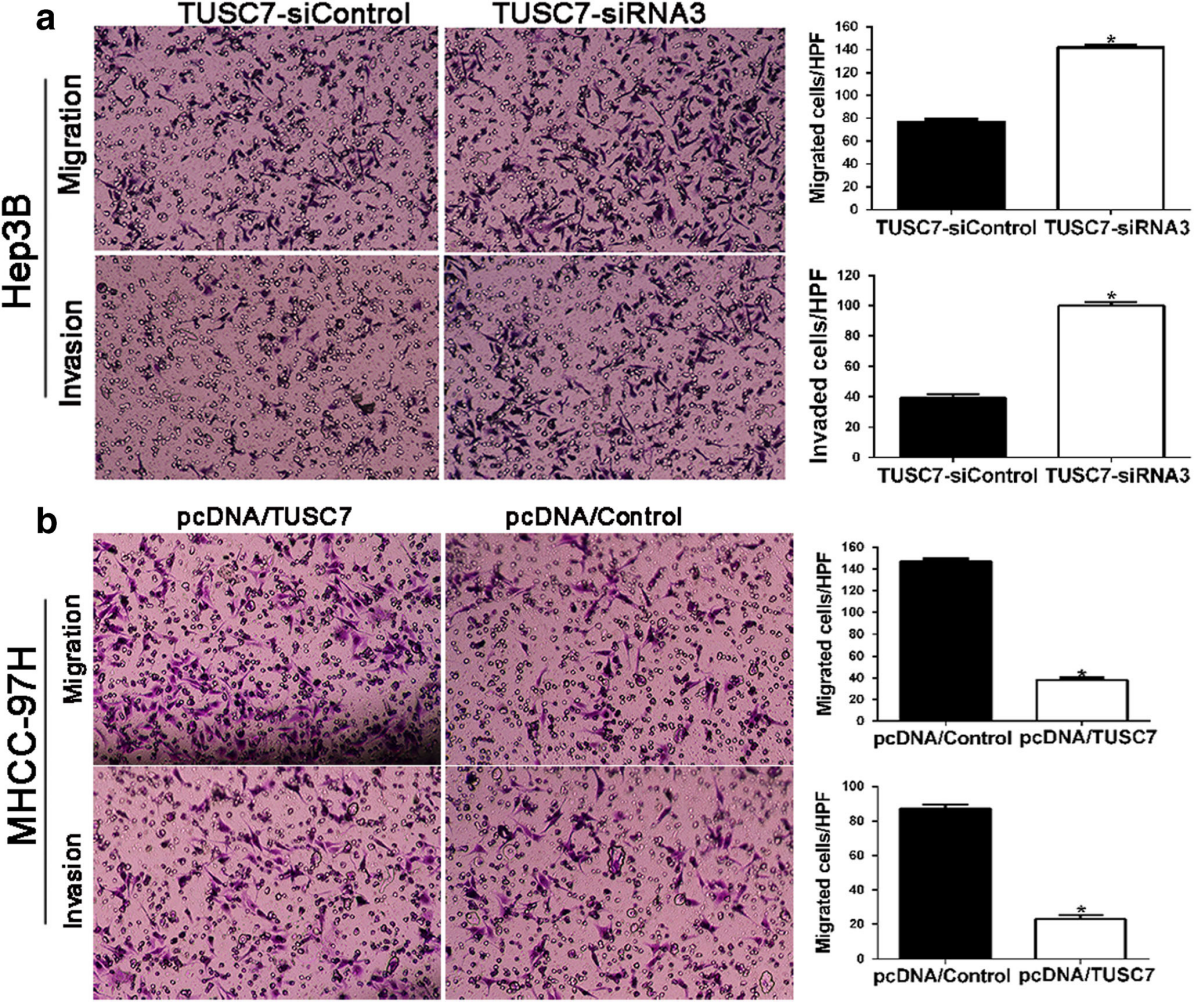
Transwell assays to assess the effect of *TUSC7* on migration and invasion in HCC cells. **a** The effect of *TUSC7* on migration and invasion ability in Hep3B cells. **b** The effect of *TUSC7* on migration and invasion ability in MHCC97H cells. *P < 0.05, by *t* test

To explore the biological significance of *TUSC7* in HCC progression, we manipulated *TUSC7* levels in HCC cells and examined the alteration of the metastatic behavior of HCC cells. Firstly, we used *TUSC7*-siRNAs (*TUSC7*-siRNA1, *TUSC7*-siRNA2, and *TUSC7*-siRNA3) to downregulate the expression of *TUSC7* in Hep3B cells. Additionally, pcDNA/*TUSC7* vector and pcDNA/Control vector were transfected into MHCC97H cells. The result of qRT-PCR revealed that *TUSC7*-siRNA3 was the most effective siRNA which inhibited the expression of *TUSC7* in Hep3B cells significantly (*p* < 0.05, Fig. 3a). Then, we found that downregulation of *TUSC7* resulted in increased migration and invasion of Hep3B cells (Figs. 3b and 4a). Conversely, the pcDNA/*TUSC7* vector significantly upregulated the levels of *TUSC7* in MHCC97H cells (*p* < 0.05, Fig. 3a) and resulted in diminished migration and invasion of MHCC97H cells (Figs. 3c and 4b). These data indicated that *TUSC7* can inhibit migration and invasion of HCC cells.

### *TUSC7* suppresses EMT in HCC

**Fig. 5:**
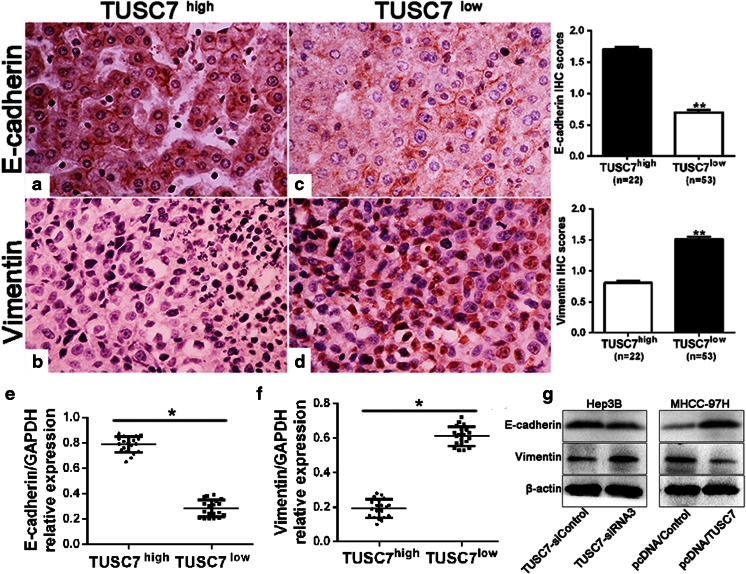
*TUSC7* inhibited EMT progression in HCC. *a*–*d* Immunohistochemistry staining of E-cadherin and vimentin in HCC tissues. In cases of high *TUSC7* expression tissue group (*a*, *b*), there was strong E-cadherin and no detectable vimentin protein expression in the same tissue section. In contrast, in the cases of low *TUSC7* expression tissue group (*c*, *d*), there was no detectable E-cadherin and strong vimentin protein expression. Values are depicted as mean ± SD; **p < 0.001, by *t* test. *Scale bar* = 100 μm. *e*, *f* Expression of EMT mRNA markers was assessed by qRT-PCR in the low *TUSC7* expression tissue group (≤0.33, *n* = 38) and high *TUSC7* expression tissues group (>0.33, *n* = 37), both groups from HCC samples. *g* Hep3B and MHCC97H cells with different *TUSC7* levels were subjected to western blot for E-cadherin and vimentin. Representative western blot showed that downregulation of *TUSC7* obviously increased protein expression of vimentin and reduced E-cadherin expression in HCC cells. *P < 0.05, by *t* test

It is well recognized that EMT plays a critical role in HCC cell migration and invasion [37]. Therefore, we explored whether *TUSC7* had effects on EMT of HCC. Firstly, we respectively analyzed the correlation of expression levels of *TUSC7* and E-cadherin as well as *TUSC7* and vimentin in 75 paired HCC tissues and adjacent non-tumor tissues by immunohistochemical staining. We found that E-cadherin expression was distinctly repressed and vimentin expression was notably increased in the low *TUSC7* tissues group compared to that in the high group (Fig. 5(a–d)). Moreover, the results were confirmed by qRT-PCR in HCC tissues (Fig. 5(e, f)) and western blot in HCC cells (Fig. 5(g)). Therefore, we conclude that *TUSC7* inhibited EMT in HCC.

### miR-10a is a downstream target of *TUSC7*

**Fig. 6:**
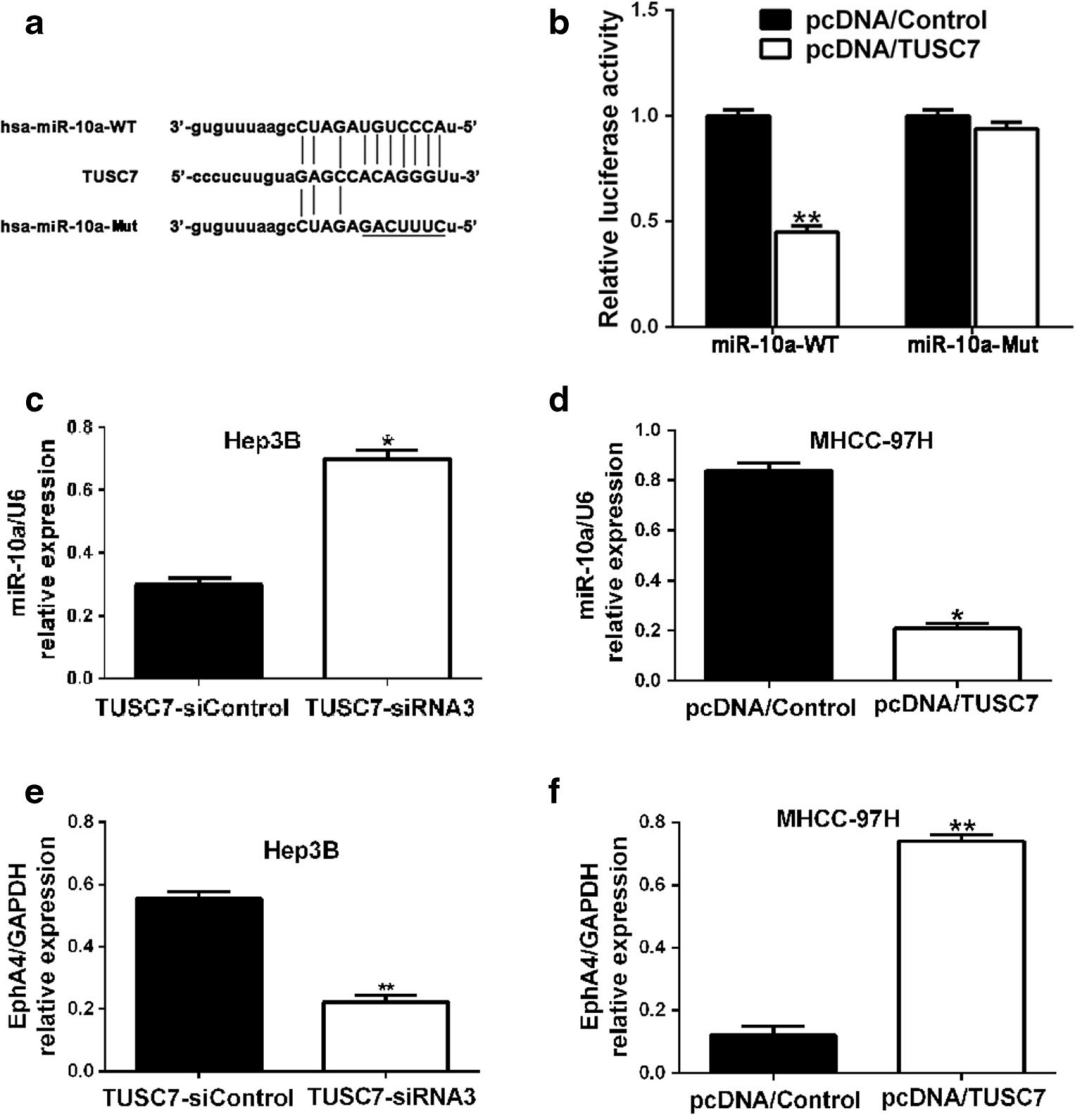
*TUSC7* targets miR-10a. **a** *TUSC7* binding sequence in miR-10a-WT and sequence of miR-10a-Mut. **b** *TUSC7* overexpression significantly suppressed the luciferase activity that carried wild-type but not mutant-type miR-10a. And *TUSC7* overexpression almost had no effect on the luciferase activity that carried neither wild-type nor mutant-type miR-10a. *n* = three repeats with similar results; **p < 0.01, by *t* test. **c**, **d** qRT-PCR revealed that *TUSC7* could negatively regulate miR-10a expression; **e**, **f** qRT-PCR revealed that *TUSC7* could positively regulate the mRNA expression level of EphA4. *P < 0.05, by *t* test

As we have mentioned before, recent studies show that *TUSC7* may function as a competing endogenous RNA (ceRNA) or a molecular sponge by modulating the biological functions and concentration of miRNAs in cancers [20, 38]. To investigate the potential downstream miRNAs of *TUSC7* and their interactions in HCC, bioinformatics tools (MicroRNA, Mircode, Starbase v2.0, and RNAhybrid) were used to analyze the potential complementary base pairing between *TUSC7* and miRNAs. The result revealed that dozens of miRNA binding sites were present in *TUSC7* (data not shown). We found that miR-10a contained the complementary sequence of *TUSC7 *(Fig. 6a). Additionally, our results have shown that *TUSC7* could repress EMT progression of HCC (Fig. 5(a–g)) and miR-10a has been reported to facilitate EMT in HCC [32]; we then focused on miR-10a. To further investigate whether miR-10a was a functional target of *TUSC7*, the dual-luciferase reporter assay was performed. We found that co-transfection of pcDNA/*TUSC7* and miR-10a-WT strongly decreased the luciferase activity while co-transfection of pcDNA/Control and miR-10a-WT did not change the luciferase activity (Fig. 6b), suggesting that miR-10a was a target of *TUSC7*. In parallel, we constructed a reporter plasmid where the *TUSC7* seed region binding site was mutated (miR-10a-Mut) to test binding specificity (Fig. 6a). Consequently, co-transfection of pcDNA/*TUSC7* and miR-10a-Mut did not change luciferase activity (Fig. 6b). Thus, these results demonstrated that *TUSC7* could directly bind to miR-10a at the miRNA recognition site.

To further confirm whether *TUSC7* exerted its function through miR-10a, we determined the expression levels of miR-10a in Hep3B cells transfected with *TUSC7*-siRNA3 and in MHCC97H cells transfected with pcDNA/*TUSC7*. The qRT-PCR results revealed that miR-10a expression was visibly elevated in Hep3B cells transfected with *TUSC7*-siRNA3 and clearly reduced in MHCC97H cells transfected with pcDNA/*TUSC7* (Fig. 6c, d, respectively). It has been reported that miR-10a could promote EMT in HCC through the miR-10a/*EphA4* axis [32]. Our data showed that ectopic expression of *TUSC7* could affect the messenger RNA (mRNA) levels of *EphA4* in HCC cells (Fig. 6e, f), which further confirmed that miR-10a is a target of *TUSC7* in HCC. Taken together, these data suggest that *TUSC7* might repress EMT through the *TUSC7*-miR-10a-*EphA4* axis in HCC.

### miR-10a reverses the inhibitory effects of *TUSC7* in HCC cells

**Fig. 7:**
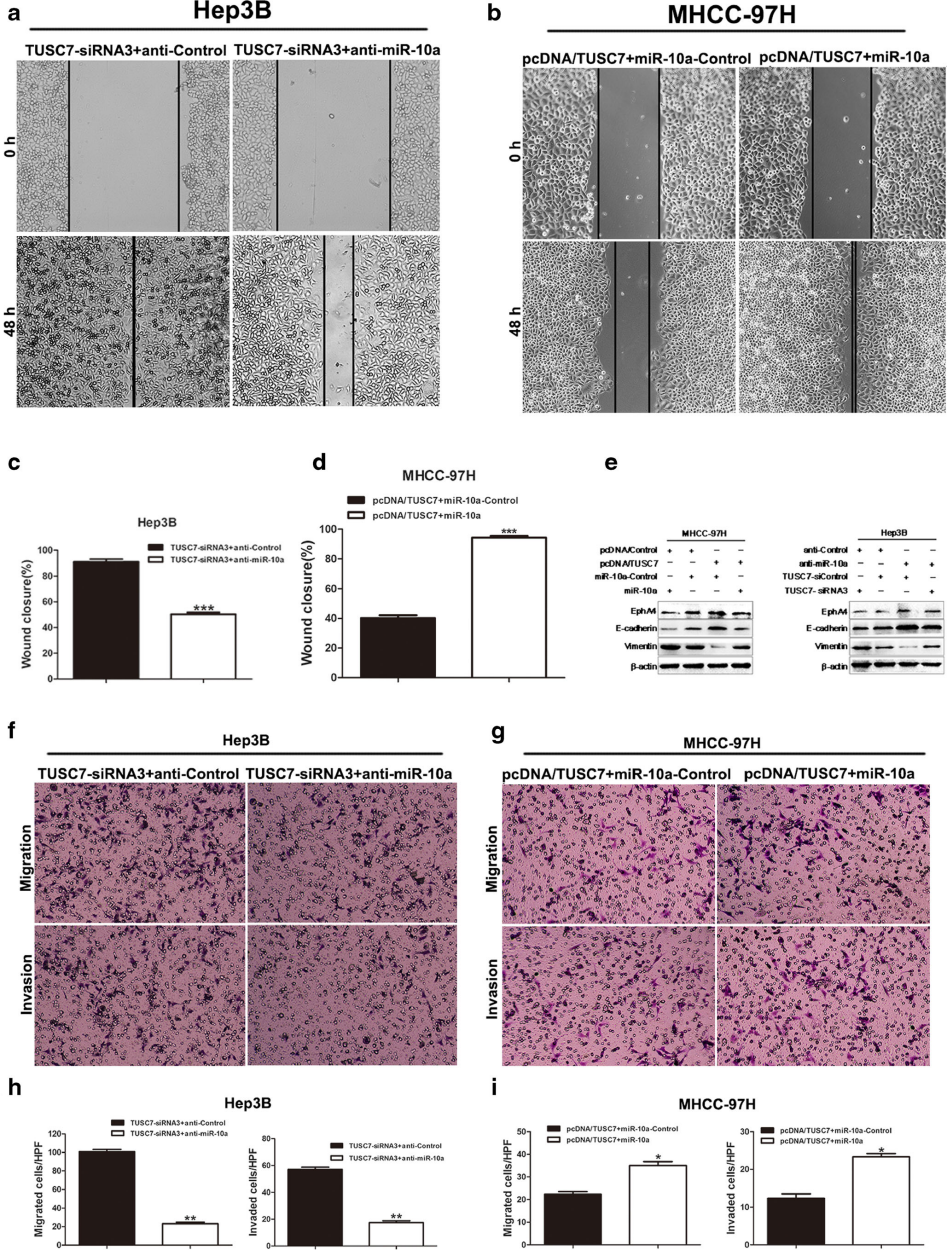
miR-10a reverses the inhibitory effects of *TUSC7* on HCC cells. **a**–**d** Wound healing assays showed that miR-10a largely reversed the inhibitory effect of *TUSC7* on cell mobility. **e** Western blot revealed that miR-10a largely reversed the inhibitory effect of *TUSC7* on EMT. **f**–**i** Transwell assays showed that miR-10a could largely reverse the inhibitory effect of *TUSC7* on cell migration and invasion.* *P* <0.05, ** *P* <0.01, *** *P* <0.001, by *t* test

Although our experiments had confirmed that miR-10a was a target of *TUSC7*, the function of miR-10a in *TUSC7*-induced inhibition in HCC cells remained unclear. And in order to confirm whether *TUSC7* could suppress EMT through the *TUSC7*-miR-10a-*EphA4* axis, the further experiments were performed. Wound healing assays (Fig. 7a–d) and Transwell assays (Fig. 7f–i) showed that miR-10a could largely reverse the inhibitory effect of *TUSC7* on HCC cell migration and invasion. Western blot also revealed that the inhibition of *EphA4* protein expression and EMT by *TUSC7* could be largely reversed by miR-10a (Fig. 7e). These results indicated that miR-10a could reverse the inhibitory effects of *TUSC7* in HCC cells and *TUSC7* could suppress EMT through the *TUSC7*-miR-10a-EphA4 axis.

## Discussion

HCC patients currently have a poor prognosis, and it is without doubt that early detection and treatment could significantly increase their chances of survival. Recently, lncRNAs have shown great therapeutic potential for human diseases, including HCC [39]. For example, studies from Yuan SX et al. have revealed that *DANCR* increases stemness and offers a potential prognostic marker, and a therapeutic target, for HCC [40]. Research from Chen CL et al. unveiled the molecular mechanisms of how *PTENP1* repressed the tumorigenic properties of HCC cells and demonstrated the potential of the *SB-BV* hybrid vector for *PTENP1* lncRNA modulation and HCC therapy [41]. Accordingly, *TUSC7* was identified as a robust suppressor of cancer [21]. In this study, we found that *TUSC7* expression in HCC was significantly downregulated. *TUSC7* expression in HCC tissues was negatively associated with more tumor nodes, more venous infiltration, advanced Edmondson-Steiner grading, and advanced TNM tumor stage. Moreover, comparison of Kaplan-Meier survival curves indicated that patients with lower *TUSC7* expression in HCC tissues had notably worse prognosis. *TUSC7* was also confirmed to be an independent risk factor for HCC patients. Altogether, these clinical data suggest strongly that *TUSC7* is critical for prognosis determination in HCC patients. Furthermore, we tested the action of *TUSC7* on tumor invasion and metastasis of HCC cells by taking different approaches and found that *TUSC7* inhibited cell invasion and metastasis in HCC.

EMT, a dynamic and reversible cellular process, is characterized by a loss of cell polarity and intracellular junctions and acquirement of mesenchymal features, which could result in increased HCC cell migration and invasion [42]. Recent studies showed that lncRNAs may play critical roles in the EMT progress not only in HCC but also in other cancers [43–45]. Furthermore, it has been found that some lncRNAs could promote EMT [45, 46] while some could restrain EMT [47, 48]. For example, *lncRNA-AOC4P* has been shown to act as an HCC tumor suppressor by enhancing vimentin degradation and suppressing EMT progress [47]. Overexpression of *lncRNA-UCA1* induced EMT and increased the migratory and invasive abilities of bladder cancer cells [49]. *lncRNA-ATB* may also act on colon tumorigenesis by suppressing E-cadherin expression and promoting EMT [50]. In this study, we analyzed EMT biomarkers of HCC tissues by using immunohistochemistry and qRT-PCR and those of HCC cells by western blot. Then, we determined the expression of an epithelial marker (E-cadherin) and mesenchymal marker (vimentin) in HCC with either low or high *TUSC7* expression. Interestingly, it was found that *TUSC7* expression was positively associated with E-cadherin expression and negatively associated with vimentin expression in HCC. We concluded that TUSC7 could suppress EMT in HCC.

Growing evidence suggests that lncRNA may act as a ceRNA to regulate miRNAs in cancer progression [51]. As we have stated before, *TUSC7* acts as a tumor suppressor in human cancers by interacting with miRNAs, such as miR-23b [20] and miR-211 [38]. It has been reported that miR-10a could facilitate cell migration, invasion, and EMT by directly targeting the 3′-UTR of *EphA4* transcript to reduce its expression in HCC [32]. *EphA4* could inhibit cell migration and invasion by regulating the EMT process through the β1-integrin signaling pathway [32]. Hence, combining our previous results and the bioinformatics analysis, we focused on miR-10a and its downstream target *EphA4*. Our results showed that miR-10a was indeed a downstream target of *TUSC7*. We found that acting as a sponge of miR-10a, *TUSC7* could therefore directly interact with miR-10a to restrain its function. Thus, when the expression level of *TUSC7* was reduced, its inhibition on miR-10a would be attenuated. The expression level of miR-10a would then be increased, which could lead to decreased expression of *EphA4*. Therefore, we have confirmed that the downregulation of *TUSC7* could enhance miR-10a expression to reduce *EphA4* expression, thereby promoting migration, invasion, and EMT in HCC, at least in part.

In summary, our data indicate that *TUSC7* may function as a tumor suppressor in HCC. Mechanistically, our experimental data demonstrate that targeting the *TUSC7*-miR-10a-*EphA4* axis may represent a novel therapeutic application in HCC.
